# Potential impacts from simulated vessel noise and sonar on commercially important invertebrates

**DOI:** 10.7717/peerj.12841

**Published:** 2022-01-26

**Authors:** David M. Hudson, Jason S. Krumholz, Darby L. Pochtar, Natasha C. Dickenson, Georges Dossot, Gillian Phillips, Edward P. Baker, Tara E. Moll

**Affiliations:** 1 McLaughlin Research Corporation, Middletown, Rhode Island, United States of America; 2 Remote Ecologist, Inc., Darien, Connecticut, United States of America; 3 Center for Environmental Sciences and Engineering, University of Connecticut, Storrs, Connecticut, United States of America; 4 Research and Conservation Department, The Maritime Aquarium at Norwalk, Norwalk, Connecticut, United States of America; 5 University of Rhode Island, Kingston, Rhode Island, United States; 6 Naval Undersea Warfare Center Division, Newport, Rhode Island, United States of America; 7 Graduate School of Oceanography, University of Rhode Island, Narragansett, Rhode Island, United States of America

**Keywords:** Crustacean, Sound, Anthropogenic impact, Lobster, Crab, Anthropogenic noise

## Abstract

Human usage of coastal water bodies continues to increase and many invertebrates face a broad suite of anthropogenic stressors (*e.g*., warming, pollution, acidification, fishing pressure). Underwater sound is a stressor that continues to increase in coastal areas, but the potential impact on invertebrates is not well understood. In addition to masking natural sound cues which may be important for behavioral interactions, there is a small but increasing body of scientific literature indicating sublethal physiological stress may occur in invertebrates exposed to high levels of underwater sound, particularly low frequency sounds such as vessel traffic, construction noise, and some types of sonar. Juvenile and sub-adult blue crabs (*Callinectes sapidus*) and American lobsters (*Homarus americanus*) were exposed to simulated low-frequency vessel noise (a signal was low-pass filtered below 1 kHz to ensure low-frequency content only) and mid-frequency sonar (a 1-s 1.67 kHz continuous wave pulse followed by a 2.5 to 4.0 kHz 1-s linear frequency modulated chirp) and behavioral response (the animal’s activity level) was quantified during and after exposure using EthoVision XT™ from overhead video recordings. Source noise was quantified by particle acceleration and pressure. Physiological response to the insults (stress and recovery) were also quantified by measuring changes in hemolymph heat shock protein (HSP27) and glucose over 7 days post-exposure. In general, physiological indicators returned to baseline levels within approximately 48 h, and no observable difference in mortality between treatment and control animals was detected. However, there was a consistent amplified hemolymph glucose signal present 7 days after exposure for those animals exposed to mid-frequency sound and there were changes to *C. sapidus* competitive behavior within 24 h of exposure to sound. These results stress the importance of considering the impacts of underwater sound among the suite of stressors facing marine and estuarine invertebrates, and in the discussion of management actions such as protected areas, impact assessments, and marine spatial planning efforts.

## Introduction

Sound levels from a variety of anthropogenic activities may result in detrimental effects on marine organisms as humans’ impact on the ocean soundscape has increased over the last century (*e.g*., Slabbekoorn et al., 2010; Carroll et al., 2017; Edmonds et al., 2016). Activities such as commercial shipping, pile driving, and the use of sonar and explosives can impact fitness, particularly in invertebrate and fish species that rely on sound-dependent activities (*e.g.*, Popper & Hawkins, 2019), for which there are still profound gaps in knowledge (Popper et al., 2021).

Acoustic sources have been shown to have significant effects on a number of invertebrate species, including bivalve mollusks, cephalopods, and crustaceans (reviewed by de Soto, 2016; Solan et al., 2016; Walsh, Arnott & Kunc, 2017). Invertebrates experience sublethal impacts such as measurable stress (*e.g*., Filiciotto et al., 2014), disruption in feeding (Celi et al., 2015; Fitzgibbon et al., 2017), sluggish return to shelter or inability to evaluate shelter (*e.g*., Walsh, Arnott & Kunc, 2017), and increased energy expenditure (Wale, Simpson & Radford, 2013a; Edmonds et al., 2016). These studies all support that they may be negatively affected by low-frequency sound (10–1,000 Hertz [Hz]), but invertebrates may sense vibration and pressure waves of particle motion at these frequencies and detectably respond (*e.g.*, Popper, Salmon & Horch, 2001; Roberts & Elliott, 2017), as is evident in crustaceans (Edmonds et al., 2016; Roberts et al., 2016) and bivalves (Roberts et al., 2015). Some crustaceans have also been shown to increase their metabolic rate during exposure to ship noise (Wale, Simpson & Radford, 2013a), indicating short- to medium-term stress or tissue repair effects. These negative effects may be cumulative for species already facing population stress from fisheries.

Characterization of organisms’ exposure to the full acoustic field (including both acoustic pressure and particle motion) is necessary, as particle motion analysis has been identified to be an essential factor studying the effect of the acoustic environment on organismal responses (Popper et al., 2014; Hawkins, Pembroke & Popper, 2015; Nedelec et al., 2016; Hawkins & Popper, 2017; Hawkins & Popper, 2018; Popper & Hawkins, 2018). Certainly, field-based studies have a benefit of occurring in *in situ* conditions that make contextual sense with additional factors like substrate vibration (Roberts & Elliott, 2017), though as several reviews note, it is important to take an integrated approach to be able to characterize response thresholds (*e.g.*, Carroll et al., 2017; Roberts & Elliott, 2017). While there is difficulty in replicating natural conditions in a lab, which is a valid concern, field experiments lack the control present in a lab environment, along with the ability to more closely observe the potential physiological pathways for non-auditory impacts to invertebrates from particle motion related to high amplitude low frequency sound (*i.e.*, it can still harm you even if you can’t “hear” it) (Carroll et al., 2017). Given the challenges for field deployment of relevant gear, and the team’s desire to determine whole animal behavioral responses along with the physiological response to acoustic stress, a mesocosm setup was chosen.

This study examined the activity level and competitive changes and sublethal physiological impacts of simulated sonar and boat noise on subadult American lobster (*Homarus americanus*) and blue crab (*Callinectes sapidus*). *H. americanus* and *C. sapidus* are both commercially important and critically dependent on nearshore habitats of the eastern United States (U.S.). Studies suggest that ocean ambient noise continues to trend upward, with the predominant low frequency source being vessel noise (Haver et al., 2018; Shi et al., 2019). Regional estimates suggest sound levels have risen by approximately 3 decibels (dB) per decade (Mazzuca, 2001; Andrew et al., 2002; Committee on Potential Impacts of Ambient Noise in the Ocean on Marine Mammals, National Research Council, 2003; U.S./Worldwide: Haver et al., 2018; Shi et al., 2019). Using a laboratory tank study, changes in posture, foraging behavior, exploratory behavior, escape response, and physiology (using hemolymph glucose and heat shock protein 27 (HSP27) as a stress marker) were examined compared to a non-exposed control. Competitive interactions between *C. sapidus* and a common invasive competitor (the green crab *Carcinus maenas*) were also conducted to evaluate whether exposure to boat noise would alter how the *C. sapidus* respond to a competition scenario.

## Methods

### Husbandry

Field-collected animals were housed outdoors in ambient water and light conditions at the University of Rhode Island’s (URI) aquarium facility in Narragansett, Rhode Island. Individual plastic mesh bins were used to separate and prevent aggressive interactions and allow tracking of individual animals. Juvenile *H. americanus* (cephalothorax length ≈45–70 millimeters (mm)) were collected by the Rhode Island Department of Environmental Management Narragansett Bay ventless trap program. Juvenile *C. sapidus* (carapace width ≈50–80mm) were hand-collected in Point Judith Pond, Rhode Island. The animals were immediately transported to the URI flow-through facility, measured, and acclimated to the facility for at least 14 days. Crabs and lobsters were fed a rotating diet of frozen butterfish (*Peprilus triacanthus*), scup (*Stenotomus chrysops*), and squid (*Loligo* sp.), 3 days per week to satiation, with uneaten food removed.

### Acoustic exposure and tank characterization

**Figure 1:**
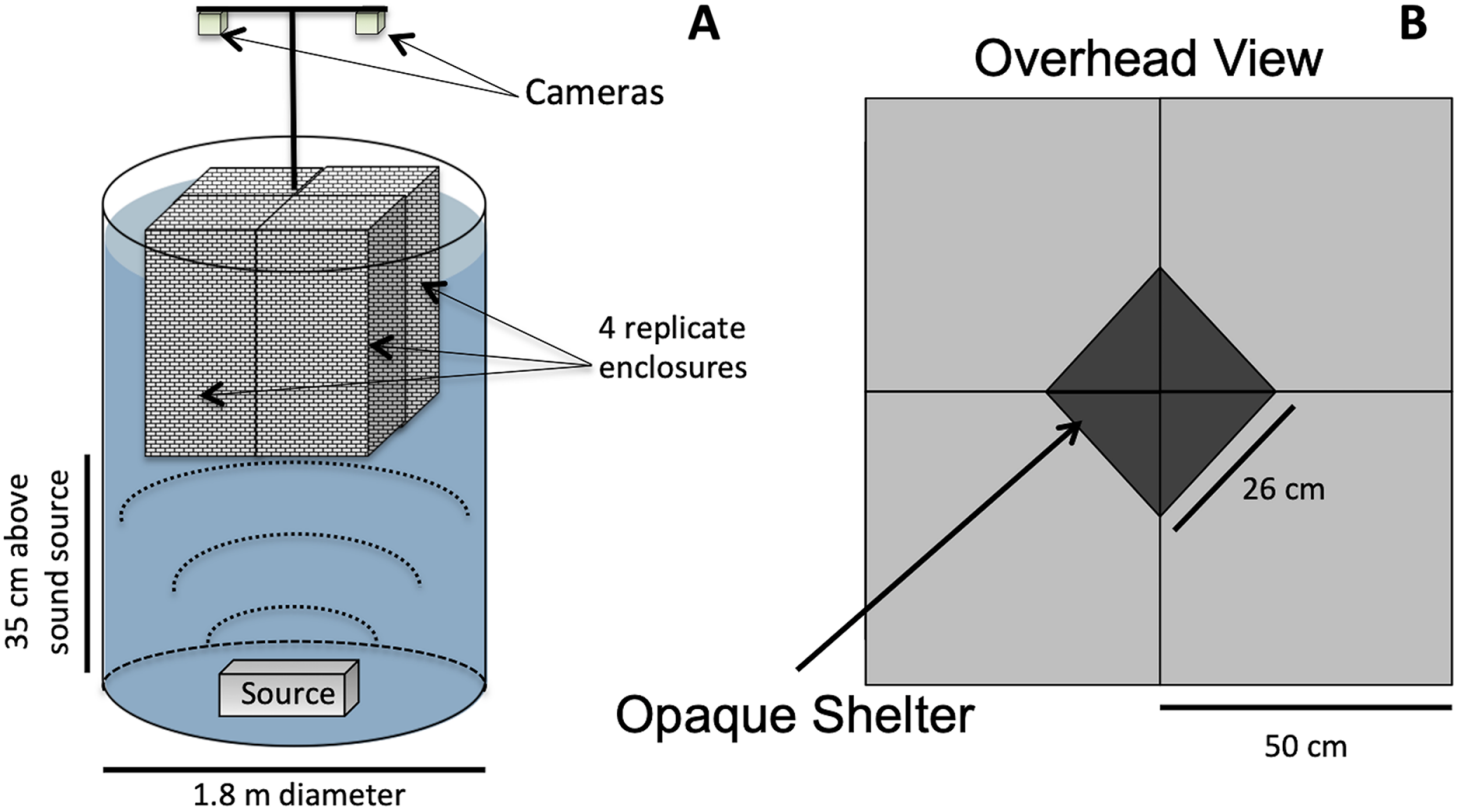
Vertical sound cylinder exposure design. The exposure setup for animals utilized a modified standing wave tube design in a vertical cylindrical tank (not to scale). (A) Overall setup for exposures, with animals isolated visually and vibrationally through including mesh in-between enclosure replicates and by keeping the mesh floor loose enough to not transmit vibrations between chambers. (B) Overhead view of shelters for four, 50 × 50 cm arenas, with a triangular shelter with a hypotenuse of 26 cm.

**Figure 2:**
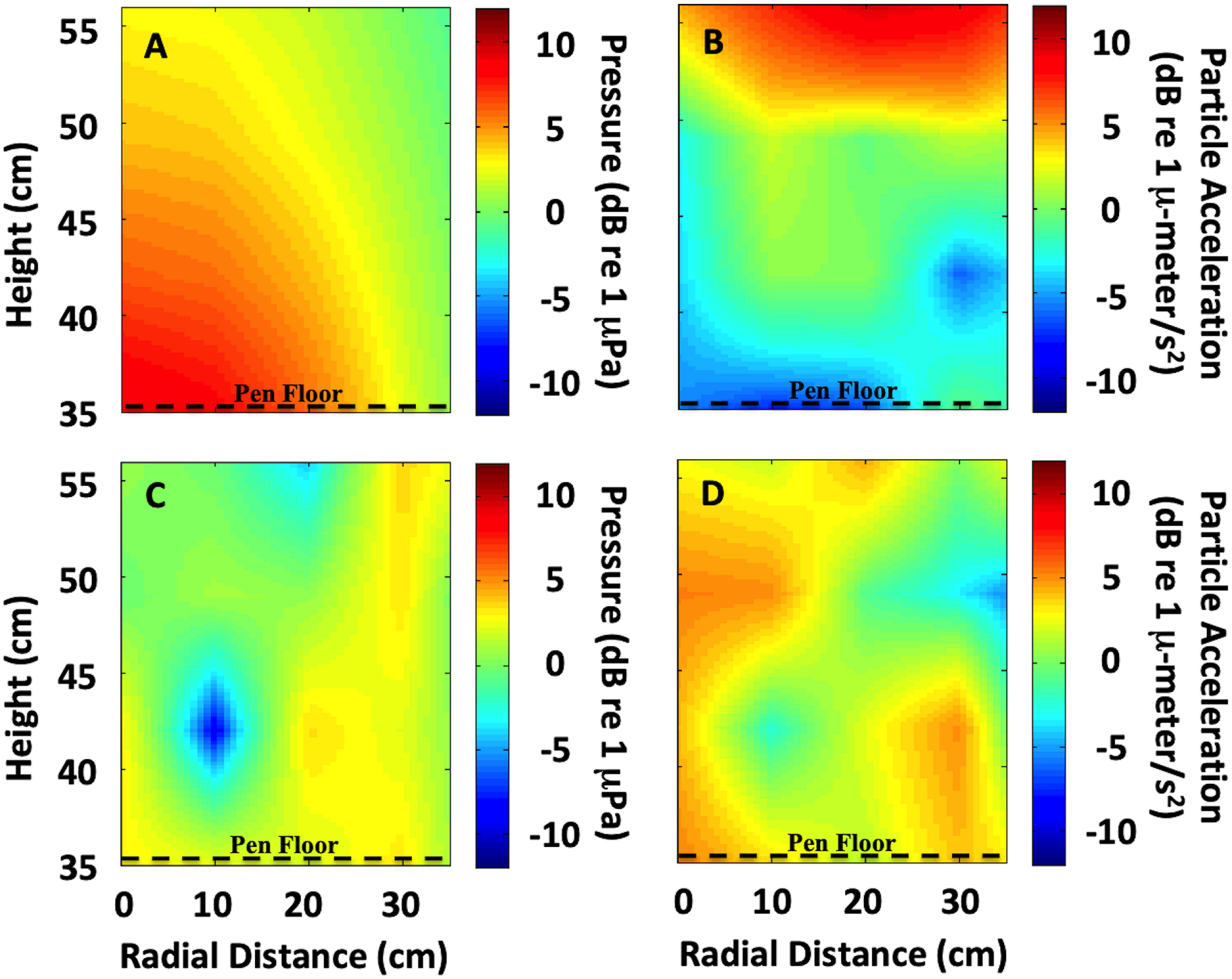
Sound field quantification in the exposure chamber. Quantification of the sound field variability (measured and interpolated) in the exposure chamber is shown, with radial distance from center over sound source (0 cm radial distance) and height (cm) over sound source. The enclosure floor was 35 cm above the sound source. • The heat map is the variance in the field, not absolute numbers. It illustrates the variation in the sound field at different heights, and what the animals were exposed to given the height of the pen floor. (A) Low frequency (<1 kHz) uniform sound field (300 Hz shown). (B) At low frequencies maximum particle motion occurs at the pressure-release surface (300 Hz shown). (C) Higher frequencies showed a more complex sound field, including nulls (4 kHz shown). (D) At higher frequencies, multiple acoustic modes exist (4 kHz shown). Units are in normalized dB re 1 µPa for sound pressure and dB re 1 µ-meter/s^2^ for particle acceleration.

Animals were exposed to either low-frequency acoustic energy (simulated vessel noise, “Boat Noise”), mid-frequency acoustic energy (simulated sonar), or ambient silence (control). Our method of acoustic exposure relied upon a laboratory tank environment (Fig. 1). While *in situ* exposure would be optimal, a laboratory exposure allows more controlled conditions and easier recording of experimental trials. However, the sound field can be difficult to control in a tank setup. Previous exposure experiments (Celi et al., 2013; Wale, Simpson & Radford, 2013a; Wale, Simpson & Radford, 2013b; Filiciotto et al., 2014; Celi et al., 2015; Walsh, Arnott & Kunc, 2017), while suitably replicating the animal’s natural environment, may have inadvertently induced an uneven sound field. A sound source positioned at the side of a tank may produce unknown artifacts due to asymmetric reflection-based interference patterns. To overcome an asymmetric sound field, the vertical cylinder exposure setup for this study was based upon a modified standing wave tube design similar to that employed in previous studies (Fay & Popper, 1974; Hawkins & MacLennan, 1976; Wysocki et al., 2007; Dossot et al., 2017), and we characterized the sound exposure field to optimize the animals placement (Fig. 2).

For this study, the sound projector was placed at the bottom center of a double-walled (5 cm) fiberglass cylindrical tank measuring 1.8 m in diameter (Fig. 1A). The overall water depth was 1.5 m. The tank size allowed for four replicate exposure pens to be placed over the sound source (Fig. 1B). The pens were made from an acoustically transparent mesh and hung from above using polypropylene line attached to a wall outside the sound field, which decoupled any unintended mechanical vibrations into the pens. The multiple enclosures permitted four simultaneous exposures per experimental iteration. The lighting was strictly controlled and animal behavior was tracked using downward-looking GoPro® cameras.

Because bioacoustic tank experiments may harbor a sound field which may be different from an animal’s natural environment (Rogers et al., 2016), our goal was to create a suitably uniform sound field over the spatial extent of the pen enclosures and also over the frequency band of interest (below 4 kilohertz (kHz)). In addition, particle motion analysis has been identified as a prerequisite for acoustic ecology going forward (Popper et al., 2014; Hawkins, Pembroke & Popper, 2015; Nedelec et al., 2016; Hawkins & Popper, 2017; Hawkins & Popper, 2018; Popper & Hawkins, 2018). To empirically characterize the sound field, acoustic measurements were taken at a 5-centimeter (cm) sampling grid along a radial cross-section of the tank, from the surface down to the top of the sound projector in the center of the tank, and over to the edge of the tank. At each measurement location a series of tones swept between 60 Hz to 4 kHz determined the frequency response of the tank. Acoustic pressure levels were measured using an Underwater Sound Reference Division (USRD) F-42 hydrophone. The acoustic particle acceleration was measured using a prototype vector sensor developed specifically for the U.S. Navy by Wilcoxen Research Inc. Both the F-42 hydrophone and the Wilcoxen vector sensor were calibrated in USRD facilities, over the band of interest, using identical mounting fixtures employed during our tank characterization measurements.

An interpolated sound field map of the tank cross-section was calculated at individual frequencies and for signals used during each treatment (Fig. 2). At low frequencies (below 1 kHz), the sound field was fairly uniform and behaved similar to a standing wave tube apparatus because only one acoustic mode was excited. For example, at 300 Hz the sound pressure level radiated evenly above the source (Fig. 2A) and the maximum particle motion occurred at the pressure-release surface (Fig. 2B). However, at higher frequencies (above 1 kHz), the sound field became more complex because multiple acoustic modes existed within the tank (Figs. 2C and 2D). At frequencies above 1 kHz, a true standing wave tube would be too small to meet the spatial requirements for the animal husbandry aspects of the experiment. Therefore, at these higher frequencies, our goal was to create as uniform a sound field as possible and quantify any acoustic variability.

Both low- and mid-frequency exposure signals were played through an audio player for 50 min after a 10-min acclimation period. The 50-min period was divided into 10 min of ambient silence, 30 min of sound exposure, and 10 min of ambient silence. This method allowed for comparison of behavior before, during, and after sound exposure. Control animals were handled identically, but were only exposed to ambient silence.

The low-frequency treatment simulated merchant vessel noise by employing a recording downloaded from the URI Discovery of Sound in the Sea (DOSITS, 2016) audio gallery, which consisted of broadband energy with significant mechanical-borne harmonics (*e.g*., 60 Hz). The signal was low-pass filtered below 1 kHz (to ensure low-frequency content only) and transmitted from a USRD J-11 acoustic projector. Sound pressure levels were measured between 169–172 dB referenced to 1 microPascal (re 1µPa) over the 60 Hz to 1 kHz band (over the pen volume). Particle acceleration variability was measured to be within 6 dB re 1 µ-meter/s^2^ over the pen floor’s spatial extent as it was suspended in the water column. This exposure was approximately representative of a close pass (<500 m) by a mid-size container vessel in a coastal area.

The mid-frequency treatment consisted of two simulated sonar signals transmitted in repeated fashion. A 1-s 1.67 kHz continuous wave pulse was followed by a 2.5 to 4.0 kHz 1-s linear frequency modulated chirp, with a 1-s pause inserted between each signal. Signals were transmitted from a Lubell Labs® source. Sound pressure levels were measured between 177–182 dB re 1 µPa over the pen volume. While some sonar signals can be well in excess of 200 dB re 1 µPa at 1 m, this exposure is representative of anticipated received levels similar to testing and calibration activities often performed by the Navy. For the 1.67 kHz signal, particle acceleration variability was measured to be within 8 dB 1 µ-meter/s^2^ over the pen floor’s spatial extent. It is worthwhile to note that broadband nature of the chirp signal served to level out the high-frequency specific variability identified during the tank characterization.

### Behavioral observations and physiological analysis

Animals in the stress portion of the study were acclimated for 7 days in the flow-through seawater system, then exposed (1 h) to either control (no noise, but in the experimental enclosure), boat noise, or simulated sonar (Fig. 1A). Experimental enclosures were 50 × 50 cm that included a triangular shelter of approximately 312 cm^2^ in one corner (Fig. 1B). The animals were monitored for acute activity changes and shelter use changes through analysis of experiment videos in EthoVision® XT, and for physiological changes at 0, 1, 3, and 7 days post exposure through extraction of hemolymph from the branchial cavity of crabs and lobsters using a 20 gauge needle. Hemolymph was then measured for glucose levels using an Invitrogen™ Amplex Red Glucose test kit, and for HSP using an Invitrogen Qubit kit for total protein and a Sigma-Aldrich ELISA kit for HSP27. As mentioned earlier, HSPs are effective in measuring stress in whole animal homogenates or brain tissue in other work, though it is compared here for use in hemolymph, a non-destructive sampling method. All assays were measured on a Biotek™ ELx800 plate reader.

### Competitive interactions

The impact of sound on competitive interactions was assessed using boat noise-exposed and control *C. sapidus*, introduced to similarly sized invasive *C. maenas* in the same arena described above, with the addition of a small piece of food being available in the corner opposite the shelter. *C. maenas* were chosen as the competitor since this species is present in similar numbers in the salt marshes where the *C. sapidus* were found, and has similar diet preferences, and would therefore be in direct competition with them.

Only the *C. sapidus* were exposed in this situation since they are broadly distributed in the water column, through both deep channels and the intertidal during high tide while *C. maenas* are more commonly found in shallow, intertidal areas. As boat noise at potentially damaging levels to crustaceans (*e.g*., >130 dB re 1 µPa, as reviewed by Edmonds et al., 2016) may not propagate far in water, though not enough data are available to make that conclusion, this could be a stressor affecting competitive interactions. Competition trials were performed at 24 h after exposure conducted in the same way as the previous experiment. Animals were acclimated to experimental conditions for 1 h in an adjacent holding tank, then monitored during a 1 h trial during which video was recorded *via* GoPro® cameras and behaviors categorized with EthoVision® XT. Since these are portunid (swimming) crabs that are found out on open substrate, behaviors were categorized as dominant (aggressive) behaviors (fighting instigator, food handling, food defense, merals spread, touch/push), submissive (active avoidance) behaviors (burrowing/burrowed, shelter use, rapid escape), or neutral (no clear dominant/submissive tendency) behaviors (climbing, being a victim/defender of attack, sitting in the open). Total seconds each crab performed each behavior was calculated and was then modeled using a Bayesian Linear Mixed Model, using an offset of total video time, to determine statistical differences (see below). Percent time spent was also calculated for all behaviors and was visualized as a donut plot (Wickham, 2016).

### Statistical analysis and modeling

Physiological and behavioral response data were statistically modeled using linear mixed models (LMM) and Bayesian linear mixed models (Bayesian LMM) in R Studio (R Core Team, 2020). We included animal grouping as a random term for all LMMs in order to account for any potential pseudoreplication effects. To meet all assumptions and to avoid singularity, data from the animals’ HSP and hemolymph glucose response, along with resource competition, were modeled using Bayesian LMM (Chung et al., 2013; Lüdecke et al., 2020). For the resource competition models, in addition to the grouping parameter, *C. sapidus–C. maenas* pairing were also included as a random term. The LMMs were utilized to test changes in animal activity level (velocity) and shelter use, as these models did not have issues of singularity once random effects were included (Bates et al., 2015). For all models, model fit was determined (Lüdecke et al., 2021), performance was checked (Lüdecke et al., 2021), and output was visualized (Breheny & Burchett, 2017). All models met all model assumptions.

## Results

### Behavioral observations and physiological analysis

Neither activity level (velocity) nor shelter use in *C. sapidus* and *H. americanus* varied significantly by exposure to either the mid-frequency or low-frequency treatments (LMM). However, there was a difference between the overall exploratory activity by species, with lobsters being more active than blue crabs (LMM, Conditional R^2^ = 0.299, Species Fixed Effect Estimate = 11.346, Standard error = 5.001, t-value = 2.269, *p* = 0.04263).

**Figure 3:**
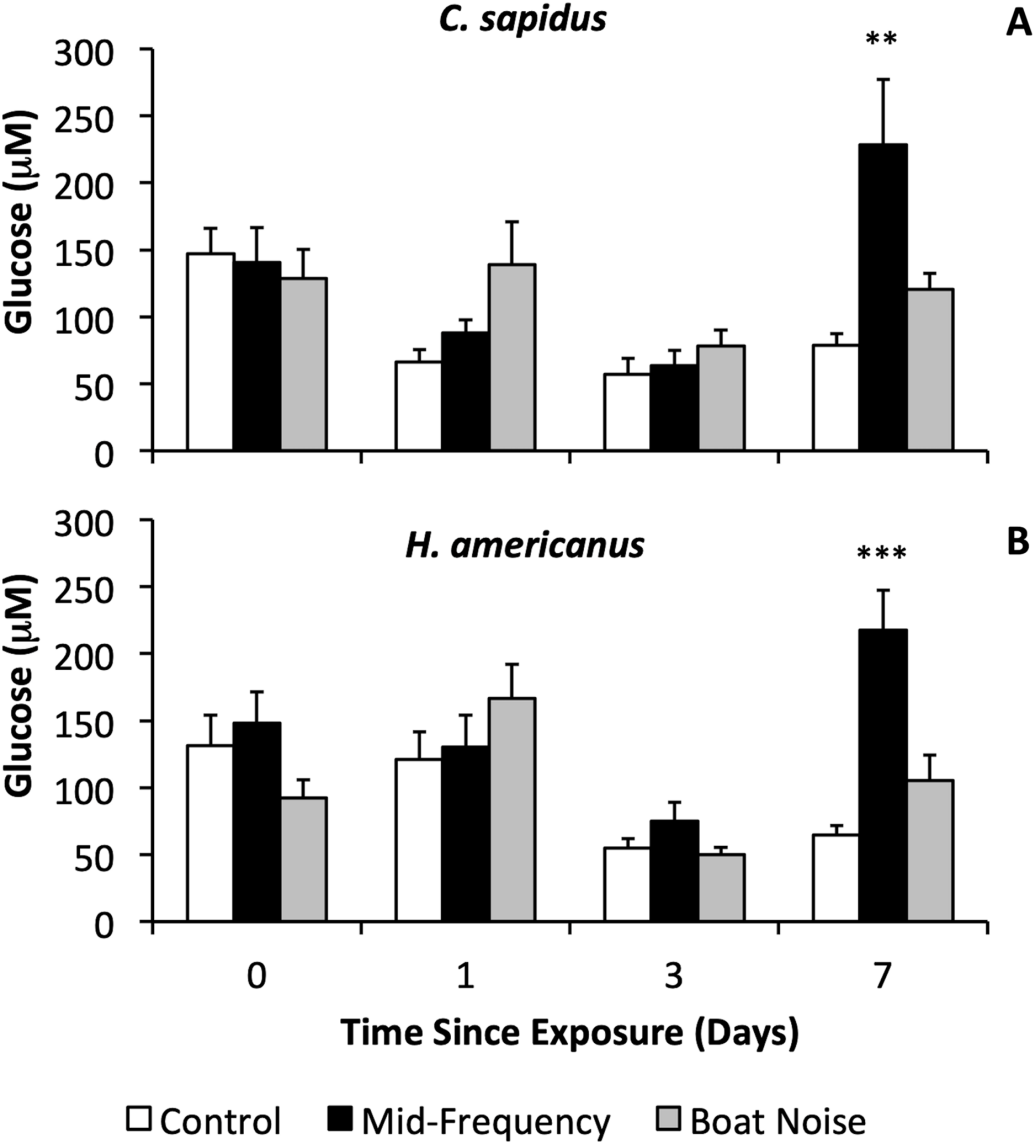
Sound treatment effects on hemolymph glucose levels in *C. sapidus* and *H. americanus*. Hemolymph glucose levels are depicted with means and standard errors by treatment (control, simulated mid-frequency sonar, or boat noise) in *C. sapidus* (A) and *H. americanus* (B). Significance is reported *versus* control based on the results of the Bayesian LMM (**p < 0.01, ***p < 0.001, error bars are standard error).

**Figure 4:**
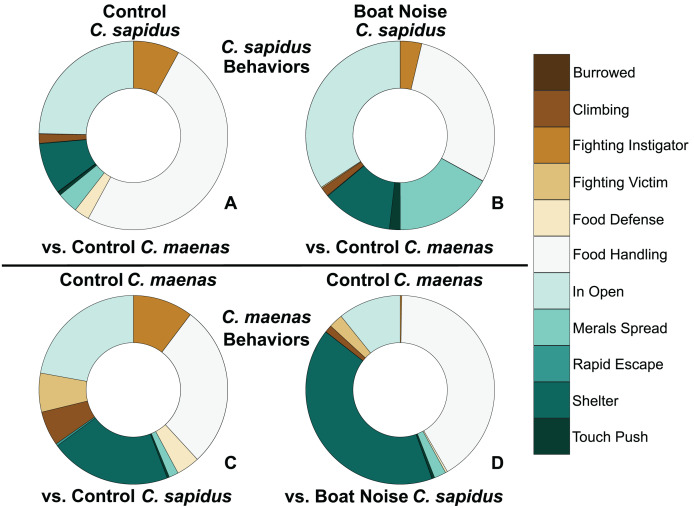
Proportion of behavioral allocation of competing *C. sapidus* and *C. maenas* during interspecific interactions when the *C. sapidus* individual was either unexposed or boat noise-exposed. The behavioral allocation proportion of dominant, neutral, and submissive behaviors are shown for the *C. sapidus*/*C. maenas* interaction. The *C. sapidus* individuals (upper two panels) in the interaction were either unexposed to noise or exposed to boat noise, while the *C. maenas* were all unexposed (lower two panels). The proportion of behavioral allocation is depicted for: (A) unexposed *C. sapidus* in competition with unexposed *C. maenas* (*N* = 34); (B) boat noise-exposed *C. sapidus* in competition with unexposed *C. maenas* (*N* = 23); (C) unexposed *C. maenas* in competition with unexposed *C. sapidus* (*N* = 34); and (D) unexposed *C. maenas* in competition with boat noise-exposed *C. sapidus* (*N* = 23).

With respect to physiological indicators of stress, there were significant differences in the stress levels indicated by hemolymph glucose after 7 days in both species (Figs. 3A, 3B, Figs. S1A, S2B), whereas HSP27 did not indicate any differences between treatments (Bayesian LMM). Both species showed significantly elevated glucose levels *vs*. controls 7 days after exposure to mid-frequency simulated sonar (Fig. S1A, *C. sapidus*, Bayesian LMM, Conditional R^2^ = 0.429, Mid-Frequency Sonar Fixed Effect = 151.85, Standard error = 48.17, t-value = 3.152, *p* = 0.001619; Fig. S1B, *H. americanus*, Bayesian LMM, Conditional R^2^ = 0.564, Mid-Frequency Sonar Fixed Effect = 152.73, Standard error = 37.74, t-value = 4.047, *p* = 5.1906 × 10^−5^) despite showing no immediate behavioral reaction to this treatment, and no elevation of glucose in earlier (24 and 72-h) samples (Figs. 3A, 4B). Other than the significant difference in glucose levels 7 days post exposure between control and mid-frequency simulated sonar exposed animals, no other time periods were significant.

### Competitive interactions

**Figure 5:**
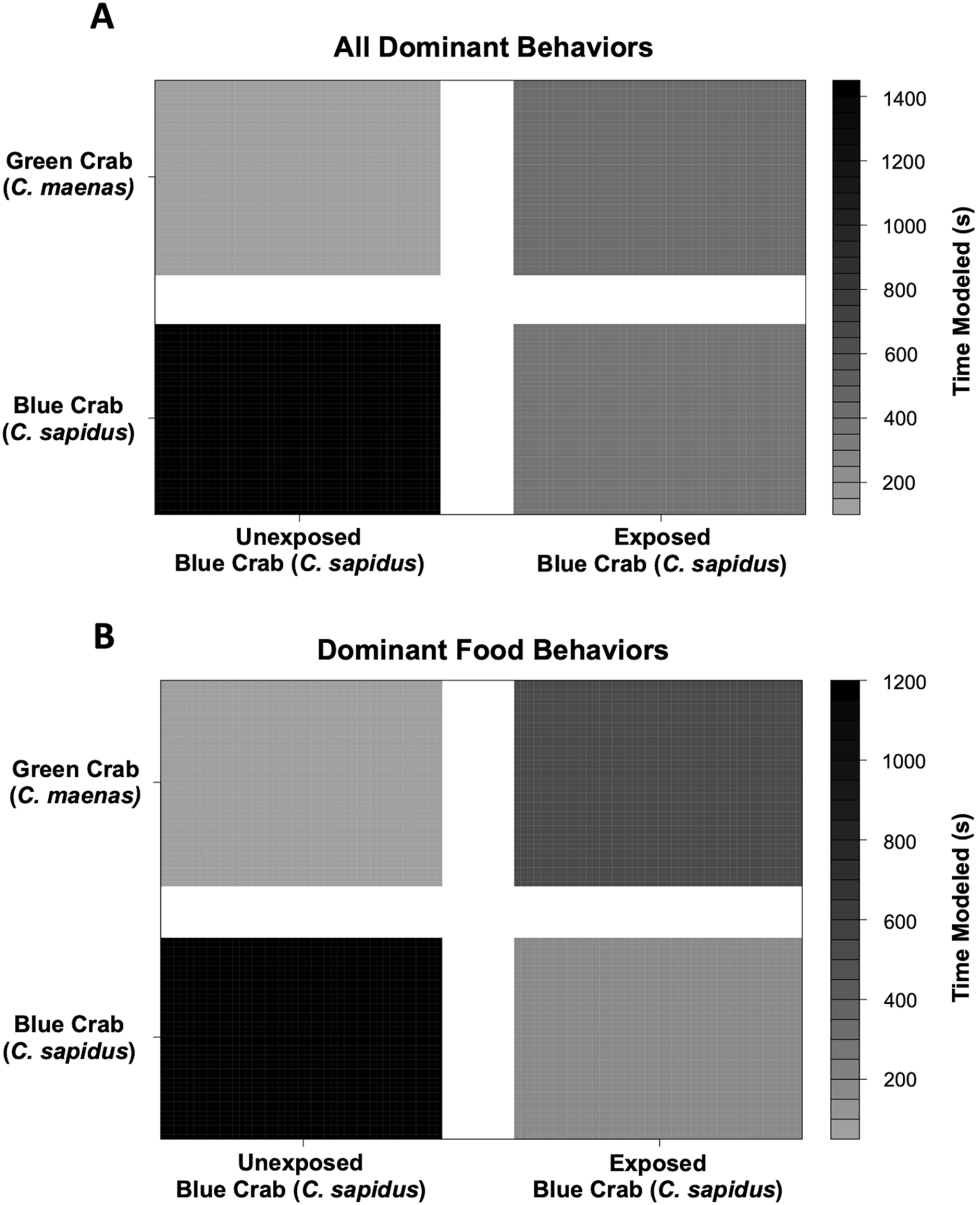
Changes in modeled dominant competitive behaviors of unexposed and noise-exposed blue crabs (*C. sapidus*) and paired unexposed green crabs (*C. maenas*). Dominant behaviors were modeled using a Bayesian Linear Mixed Model to determine the effects of and submissive competitive interactions are shown for how unexposed (Control) and boat noise-exposed (Exposed) *C. sapidus* behaved against conspecific competitors or heterospecific competitor (*C. maenas*) crabs. (A) Overall dominant behaviors modeled by time spent: changes in modeled time spent (seconds) to overall dominant behaviors in the unexposed *C. maenas* paired with unexposed and boat noise-exposed *C. sapidus* are depicted in the upper two quadrants, and changes in modeled time spent (seconds) to overall dominant behaviors in the unexposed and boat noise-exposed *C. sapidus* paired with unexposed *C. maenas* are depicted in the lower two quadrants. (B) Overall food behaviors modeled by time spent. Depicted in the same manner as (A).

Behavioral impacts on the competitive ability of *C. sapidus* persisted at 24 h post-exposure to simulated boat noise (the low-frequency treatment), and a change in behavior was also noted in its one-on-one competitor, an unexposed green crab, *C. maenas*, in the allocation of time for different activities during these trials. These changes were indicated by a change in allocation of time for a variety of behaviors coded from the video footage (Fig. 4). The behaviors observed for *C. sapidus* and *C. maenas* were related to each other, for instance *C. maenas* were frequently fighting victims of *C. sapidus* if they were exhibiting food defense behaviors. When *C. sapidus* were exposed to boat noise they displayed a decrease in time allotted to food handling, food defense, and instigating fights (Figs. 4A, 4B) and an increase in sitting in the open, rapid escape, and merals spread (an aggressive claw display). Conversely, the *C. maenas* competing with a boat noise exposed *C. sapidus* (Fig. 4D), exhibited a decreased its time fighting (all fighting behaviors), food defense, time in the open, and climbing (Figs. 4C, 4D) compared to *C. maenas* competing with an unexposed *C. sapidus*. When each behavior was modeled as a Bayesian LMM, the behaviors of pooled dominant and dominant food significantly declined for *C. sapidus* exposed to boat noise and increased for competing *C. maenas* (Fig. 5; Pooled Dominant Bayesian LMM–Conditional R^2^ = 0.732; Dominant Food Bayesian LMM–Conditional R^2^ = 0.664). For both Bayesian LMMs, the fixed factors of species (Pooled Dominant–ß = −1299.0 ± 376.7, t-value = -3.449, *p*-value = 0.000563; Food Dominant–ß = −1112.1 ± 332.9, t-value = −3.340, *p*-value = 0.00083) and the interaction between species and treatment (Pooled Dominant–ß = 1378.8 ± 575.4, t-value = 2.396, *p*-value = 0.0165; Food Dominant–ß = 1479.0 ± 508.5, t-value = 2.908, *p*-value = 0.00364) were significant, while the fixed factor of treatment alone (Pooled Dominant–ß = −1035.1 ± 1197.7, t-value = −0.864, *p*-value = 0.387; Food Dominant–ß = −1016.9 ± 619.4, t-value = −1.345, *p*-value = 0.251) was not significant. In the Bayesian LMM for neutral and submissive behaviors, there were no significant differences between treatments, species, and their interaction.

## Discussion

Anthropogenic sound and sound in general are documented to have a negative effect on fitness of animals aside from physiological stress, particularly by increasing vulnerability to predation (Simpson et al., 2016). Ambient noise level in the oceans has increased by as much as 12 dB over the last three decades, with increases in anthropogenic use, and is expected to continue to rise (Committee on Potential Impacts of Ambient Noise in the Ocean on Marine Mammals, National Research Council, 2003; Hildebrand, 2009; Haver et al., 2018; Shi et al., 2019). In terms of chronic effects, crabs exposed to repeated boat noise over time (though of an uncharacterized sound field, so perhaps not representative) experienced an increase in stress measures like metabolic rate (Wale, Simpson & Radford, 2013a), and fishes exposed to long-term sound exposure show detriments in normal behaviors, desensitization to important environmental sounds, increases in physiological indicators like cortisol, and increased infection and parasitism (*e.g*., Anderson et al., 2011; Carroll et al., 2017; Hawkins & Popper, 2018). These chronic effects are linked to decreased fitness (Simpson et al., 2016), even though there is some evidence that stress indicators and responses to biologically important signals can lessen over time as animals become acclimated to chronic noise (Slabbekoorn et al., 2010; Armstrong-Smith, 2016; Pine et al., 2016). Of particular concern for chronic exposure to anthropogenic noise is the knowledge gap in vertebrate and invertebrate responses to that noise outlined recently regarding offshore wind development in New York State, USA (Popper et al., 2021). Development involving additional anthropogenic production of noise is proposed for the western North Atlantic Ocean, so additional research is urgently needed.

Literature studies comparing acute and chronic acoustic stress are limited and are conducted on animals with widely different hearing capabilities. A number of crustaceans have behavioral thresholds to water and substrate-borne vibrations when exposed between 5–410 Hz, and can likely detect high frequency sources over 300 m away (Roberts et al., 2016). Boat noise exposure in this study overlapped in this threshold range for other crustacean species, even if the mid-frequency sonar exposure did not. While neither boat noise exposure nor mid-frequency sonar exposure seemed to elucidate an initial behavioral response change, the effects of both were detectible in post-exposure measures. Boat noise exposure elucidated a behavioral change in interactions with heterospecifics that resulted in a competitive impairment of *C. sapidus* that were exposed (Figs. 4 and 5), even though it did not show a detectable physiological signal in hemolymph glucose. Similarly, the simulated mid-frequency sonar exposure, whether the animals detected it or not, could still do lasting physiological damage, as a peak in hemolymph glucose was detected at 7 days after exposure for both test species (Fig. 3, Fig. S1). This aligns with recent work on chronic damage by air gun signals in field-exposed *Jasus edwardsii* palinurid lobsters showed the animals had an impaired righting reflex that did not improve after 365 days nor after a molting event, and confirmed statocyst damage (Day et al., 2019). Other examples of chronic stress are evident in other aquatic animals of a wide variety of hearing capabilities, though stimulus variability (intermittent stimulus) seems to produce greater stress than prolonged increased noise (Nichols, Anderson & Širović, 2015; Filiciotto et al., 2017), suggesting some degree of habituation. Short-term acute stress is evident in European sea bass (*Dicentrarchus labrax*) exposed to *in situ* pile driving stress (Debusschere et al., 2016), showing notable decreased oxygen consumption and depressed lactate response. However, this study also found no chronic impacts, in terms of significant differences in long-term growth or survival, which is further supported by studies on larval fish that showed no effect of pile driving sound on mortality (*e.g*., Bolle et al., 2012; Bolle et al., 2016), indicating that the results for finfish are also variable by species since hearing capabilities vary by species (Bruintjes et al., 2016), and our understanding of how response to chronic stimulus may differ from acute stimulus is a topic for much further research and discussion. The community effects of anthropogenic noise are certainly likely to disrupt communication signals, though more attention is being given to the potential anthropogenic effects on invertebrates in reef communities (*e.g*. Ferrier-Pagès et al., 2021). It is known that boat noise exposure can cause deficits in larval invertebrate survival (Nedelec et al., 2014), disorientation (Montgomery et al., 2006) and sublethal physiological effects on invertebrate larvae have been detected weeks after exposure to anthropogenic sound (Payne et al., 2007).

The behavioral and physiological measures of stress in this study were aligned with literature methods. A significant reduction in activity and elevated stress (increased glucose) 24 h after exposure to boat noise was observed in *C. sapidus*. Further, both *C. sapidus* and *H. americanus* showed a strong response to mid-frequency sound in elevation of glucose levels after 7 days. This result was unexpected, but the fact that it was observed at similar magnitude across both species suggests that it was not by chance. Because we did not sample beyond 7 days or retain experimental animals beyond this point, it is impossible to speculate as to whether this spike was short term, or a more severe late onset physiological response that could have led to increased mortality. This finding warrants further investigation into the causal mechanism behind this observation. It is important to note that stress, as indicated by glucose levels was elevated at time zero for all treatments and decreased over time in the control treatment, perhaps indicating that handling stress and the holding conditions may be obscuring additional experimental signal. Consistent with recent literature on crustacean exposures to noise (*e.g.*, Day et al., 2019), the results suggest the presence of a stress response that could result in impairment of the animals’ competitiveness after exposure.

The behavioral shift observed in competition experiments means that a non-exposed competitor or one with a different hearing sensitivity could monopolize food or shelter resources that would be consistent with a change in competitive vigor in the exposed animal (Figs. 4, 5). The animals exposed to high amplitude noise showed an increase in aggressive behavior, a decrease in feeding behavior, and reduced locomotion during testing relative to unexposed animals. These responses to sound indicate a behavioral shift that could result in decreased fitness if persistent. Work in other crustaceans, like the crayfish *Procambarus clarkii*, did show a difference in aggressive and active behaviors during acoustic stimulus (Celi et al., 2013). Furthermore, the observed reduction in feeding among exposed adult animals could theoretically produce a delayed physiological response similar to that detected in the 7-day mid-frequency exposure samples, as is suggested for crabs (Wale, Simpson & Radford, 2013b) and lobsters (Fitzgibbon et al., 2017). Activities which either damage hearing or mask important auditory cues could have detrimental impact on survival.

Baseline levels of stress in this study were often quite high before experiments began. Similar to studies Debusschere and colleagues (Debusschere et al., 2016) completed with the European sea bass, *Dicentrarchus labrax*, in which the cortisol response was concluded to have a strong handling bias, we expected that the level of handling and hemolymph extraction required for this experimental protocol may have elevated stress even in control animals. Whole body cortisol and HSP are traditionally used to measure primary stress response in fish and crustaceans, respectively, but measuring the glycemic response through blood glucose levels, lactate, or hormones like crustacean hypoglycemic hormone are also good measures of stress in the longer term (Chang, 2005; Stoner, 2012; Powell et al., 2017). While we did not observe any effect of treatment in our HSP27 analysis, we suspect that this is because previous studies using HSP27 in crustaceans measured HSP27 in either whole animal homogenate or brain tissue, both of which are lethal methods. Our desire to analyze a time series of response, precluded lethal methods, since the sample sizes necessary to sacrifice animals at each time interval would have been prohibitive given available resources, so we attempted to modify this protocol for use with hemolymph, which did not appear to be an effective method. However, glucose hemolymph level combined with behavioral indicators provided sufficient evidence to support our overall hypothesis that simulated boat noise and mid-frequency sonar has a measurable impact on crustacean physiology and behavior. While the effect of mid-frequency sonar remains unexplored, the negative effects of simulated boat noise exposure on competitive ability in *C. sapidus* are particularly concerning, since it is competing with several invasive species along its range, including *C. maenas* as examined here, that may have different responses to that stimulus that could tip competitive balance. Since exposed *C. sapidus* were less dominant, this allowed for the behaviors of food handling and shelter use to subsequently increase in occurrence in competing *C. maenas*, where both behaviors are ideal for *C. maenas* as they prefer crevices. These competitive assessments and other behavioral measures, such as righting behavior (flipping the animal and watching time to righting response), morbidity, and reflex responses have been used in the past to indicate stress levels in crustaceans (*e.g*., Stoner, 2012). Further investigation of behavioral responses of these animals both in mesocosm competitive scenarios and field exposure studies is needed to conclude any effect on ecological interaction.

There is still a need to determine the long-term and ecological effects of acute and chronic sound exposure, both in terms of particle acceleration, and in terms of sound pressure (which are difficult to distinguish using this experimental approach) on the ecology and fitness of invertebrates, particularly those species which are important to fisheries (Edmonds et al., 2016), and thus, also suffer fisheries related mortality. Certainly, the sensitivity of crustaceans’ sensitivity to particle acceleration alone is in need of further investigation. The use of these physiological and behavioral measures of stress in crustaceans has potential for broader application beyond acoustic stress. When combined with secondary responses like measuring resting metabolic rate after exposure, which has been effectively used to measure stress in a public aquarium environment (Rose, 2009), physiological measurements could indicate potential metabolic deficits impacting fitness prior to any physical morbidity. Future studies that bring together better measures of stress and exposures of animals to a broader suite of sounds will help us to continue to better understand the large scale effects of these increasing stressors on these animals.

## Supplemental Information

10.7717/peerj.12841/supp-1 Supplemental Information 1 Modeled sound treatment effects on hemolymph glucose levels in *C. sapidus* and *H. americanus*. Hemolymph glucose levels are depicted as scatterplot, with the Bayesian LMM results as colored bars by treatment (control, simulated mid-frequency sonar, or boat noise) in (A) *C. sapidus* and (B) *H. americanus*.

10.7717/peerj.12841/supp-2 Supplemental Information 2 Raw data for glucose concentration in hemolymph of blue crabs and lobsters in the mesocosm experiment. Each data point refers to the amount of glucose in the hemolymph of individual lobsters and crabs, by hour after exposure, by exposure, and by species.

10.7717/peerj.12841/supp-3 Supplemental Information 3 Raw data for Heat Shock Protein 27 (HSP27) in hemolymph of blue crabs and lobsters in the mesocosm experiment. Each data point refers to the amount of HSP27 per volume of total protein in the hemolymph of individual lobsters and crabs, by hour after exposure, by exposure, and by species.

10.7717/peerj.12841/supp-4 Supplemental Information 4 Raw data for shelter use by blue crabs and lobsters in the mesocosm experiment. Total shelter use time is provided, by individual animal, along with the total length of the video file, species, and treatment.

10.7717/peerj.12841/supp-5 Supplemental Information 5 Raw movement data for *Callinectes sapidus*, by treatment and individual. Total movement time is provided, by individual animal, and by treatment for *Callinectes sapidus*.

10.7717/peerj.12841/supp-6 Supplemental Information 6 Raw movement data for *Homarus americanus*, by treatment and individual. Total movement time is provided, by individual animal, and by treatment for *Homarus americanus*.

## Additional Information and Declarations

### Data Availability

The following information was supplied regarding data availability: The raw animal activity data (for *C. sapidus* and *H. americanus*) and the glucose levels, heat shock protein, and sheltering data are available in the Supplemental Files.
