# Supplementary figures and images for: Potential impacts from simulated vessel noise and sonar on commercially important invertebrates
Source: PeerJ. 2022 Jan 26;10:e12841. doi: 10.7717/peerj.12841 (PMC8800386; doi:10.7717/peerj.12841)

**A*****C. sapidus***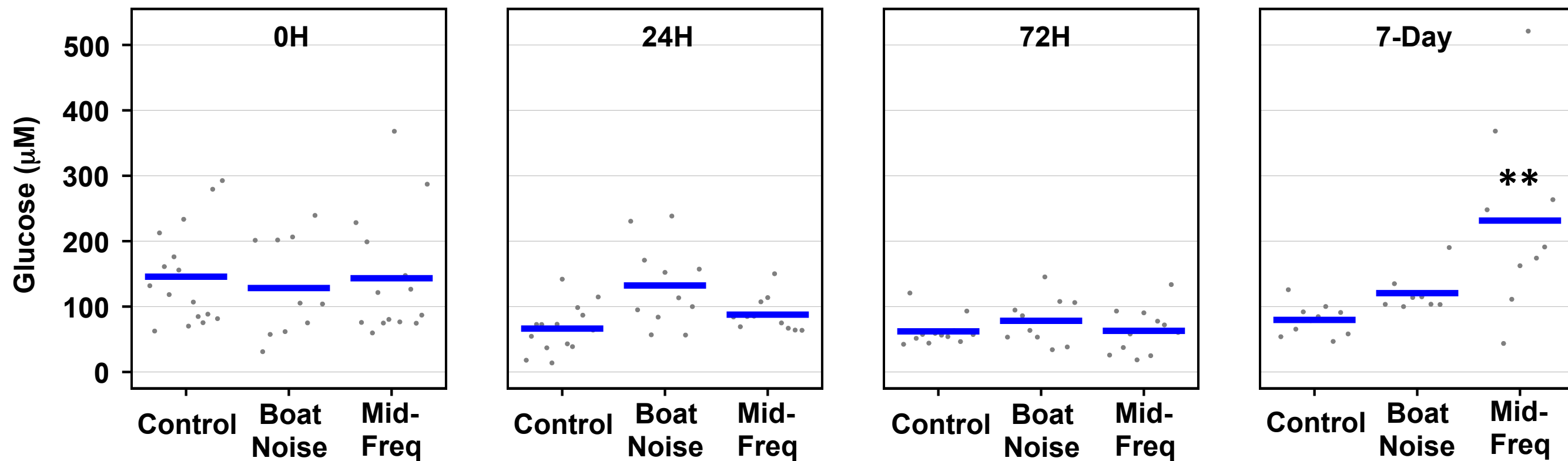**B*****H. americanus***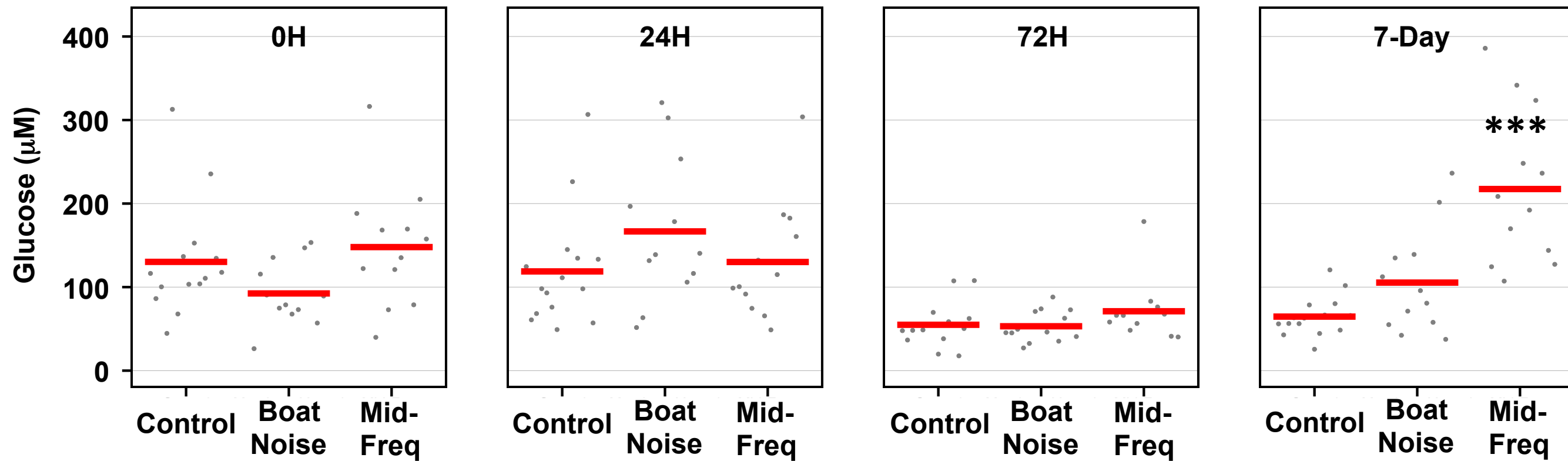

Supplement: Supplemental Information 1 — Hemolymph glucose levels are depicted as scatterplot, with the Bayesian LMM results as colored bars by treatment (control, simulated mid-frequency sonar, or boat noise) in (A) C. sapidus and (B) H. americanus. [file peerj-10-12841-s001.pdf]
